# High level of serum apolipoprotein A-I is a favorable prognostic factor for overall survival in esophageal squamous cell carcinoma

**DOI:** 10.1186/s12885-016-2502-z

**Published:** 2016-07-21

**Authors:** Xue-Ping Wang, Xiao-Hui Li, Lin Zhang, Jian-Hua Lin, Hao Huang, Ting Kang, Min-Jie Mao, Hao Chen, Xin Zheng

**Affiliations:** Department of Laboratory Medicine, State Key Laboratory of Oncology in South China, Collaborative Innovation Center for Cancer Medicine, Sun Yat-sen University Cancer Center, Guangzhou, Guangdong 510060 People’s Republic of China; Department of Laboratory Medicine, The First Affiliated Hospital of Sun Yat-sen University, Guangzhou, Guangdong China; Guangdong Esophageal Cancer Institute, Guangzhou, Guangdong China

**Keywords:** Apolipoprotein A-I, ESCC, Prognosis, Overall survival

## Abstract

**Background:**

Noninvasive prognostic tools for esophageal squamous cell carcinoma (ESCC) are urgently needed. Serum lipids and lipoproteins are used for the prognosis of certain diseases; however, the prognostic value of serum apolipoprotein A-I (ApoA-I) in ESCC has not been described.

**Methods:**

Pre-treatment serum lipids and lipoprotein concentrations (including ApoA-I, Apo-B, HDL-C, LDL-C, TC and TG) were analyzed retrospectively and compared between 210 patients with ESCC and 219 healthy controls. The prognostic significance of serum lipids and lipoproteins was determined by univariate and multivariate Cox hazard models in ESCC.

**Results:**

Clinical characteristics (age, sex, pT status, pN status, pM status, pTNM status, histological differentiation or alcohol index) had no influence on baseline ApoA-I level. Serum ApoA-I, HDL-C, LDL-C, and TC levels were significantly lower and Apo-B was significantly higher in ESCC patients than in normal controls. On univariate analysis, ApoA-I, alcohol index, pT status, pN status and pTNM status were associated with significantly poor survival, and ApoA-I (*p* = 0.039), alcohol index (*p* = 0.037) and pTNM status (*p* = 0.000) were identified as prognostic factors associated with shorter survival in the multivariate analysis.

**Conclusions:**

Overall survival was shorter in ESCC patients with decreased pre-treatment ApoA-I levels. Our findings suggest that serum ApoA-I level should be evaluated as a predictor of survival in patients with ESCC.

## Background

Esophageal squamous cell carcinoma (ESCC), the predominant histologic type of esophageal cancer, is one of the most lethal malignancies of the digestive system and the sixth leading cause of cancer mortality worldwide [1, 2]. Despite improvements in treatment strategies, including surgical techniques and adjuvant chemoradiation, the overall 5-year survival rate of ESCC patients treated with surgery alone is less than 20 % [3, 4]. In recent years, many serum biomarkers, such as SCC, CYFRA21-1, and CEA, have been used to predict the survival of ESCC patents. However, these markers have limited sensitivity and specificity and are not entirely reliable [5–7]. Therefore, the identification of accurate biomarkers for ESCC is necessary to improve the clinical outcome of patients.

Abnormal levels of lipids have been shown to be closely correlated with tumor progression in several cancers [8, 9]. Cholesterol synthesis is enhanced in cancer cells compared with normal cells, as tumor cells need excess cholesterol and cholesterol biosynthesis pathway intermediates to maintain a high level of proliferation [10]. Evidence for the activation of lipid metabolism in tumor cells can be provided by quantifying the products of lipid metabolism such as apolipoprotein A-I (ApoA-I) in the serum of cancer patients [11–13].

ApoA-I is a major high-density lipoprotein cholesterol (HDL-C) component in serum, which constitutes approximately 70 % of the apolipoprotein content of HDL-C particles [14]. ApoA-I can function as an enzyme cofactor, receptor ligand, and lipid transfer carrier involved in the regulation of lipoprotein metabolism [9, 15, 16]. Beyond the known functions of ApoA-I, it may indirectly promote tumor survival through the other activation. It is overexpressed in patients with recurrent head and neck squamous cell carcinoma [17]. High ApoA-I concentration is associated with increased risk of cancer, in particular head and neck squamous cell carcinoma [18], whereas the prognostic roles of ApoA-I, Apo-B, HDL-C, low-density lipoprotein cholesterol (LDL-C), total cholesterol (TC), and triglycerides (TG) remain unclear. ApoA-I is a risk and prognostic factor in nasopharyngeal carcinoma [19]; however, its prognostic value remains unclear in ESCC.

To clarify the association between lipid profiles and ESCC, this study was designed to elucidate the associations between alterations of in vivo lipid metabolism and the occurrence of ESCC by measuring the levels of a number of relevant lipids in the pre-therapy serum of patients with ESCC in comparison with those of healthy participants.

## Methods

### Patients

Between January 2007 and July 2009, 210 eligible patients (150 men and 60 women; ages 36–79 years, median 58 years) diagnosed with ESCC at the Sun Yat-sen University Cancer Center were enrolled into this retrospective study. The demographic details are described in Table 1. All of the patients met the diagnostic criteria for ESCC. Exclusion criteria were as follows: (1) patients treated with medication or taking hormone replacement therapy or any drugs known to affect lipid metabolism before serum collection; (2) patients with concomitant diseases associated with increased serum lipid and lipoprotein levels (i.e., diabetes, hyperlipidemia, or metabolic syndrome); (3) other types of malignancy. Based on medical records, the tumor differentiation grades were classified according to the World Health Organization criteria. Stage was recorded based on the American Joint Committee on Cancer guidelines (2009) [20]. All patients received treatment. Clinical information, including demographic data, pathological tumor node metastasis (pTNM) stage, smoking status, alcohol consumption, and overall survival (OS) data were available for all patients. Tobacco index was calculated as cigarettes per day multiplied by years of smoking, and patients were divided into two groups: smoking and nonsmoking. Alcohol index was assessed as drinking or not drinking. A total of 219 healthy participants (137 men and 82 women; ages 23–79 years, median 54 years) were recruited from the physical examination department at Sun Yat-Sen University Cancer Center.

**Table 1:** The levels of ApoA-I between the ESCC patients and healthy controls

Characteristics	ESCC		*p* Value	Controls		*p* Value
	Numbers	Median (range)		Numbers	Median (range)	
Age, years			0.973			0.733
<58	102	1.21(0.83–1.64)		105	1.56(0.8–2.32)	
≥58	108	1.21(0.71–2.05)		114	1.59(1.23–2.11)	
Sex			0.084			0.887
Male	150	1.16(0.71–2.05)		137	1.56(0.8–2.24)	
Female	60	1.25(0.81–1.89)		82	1.58(1.05–2.32)	
pT status			0.175			
pT 1	17	1.15(0.95–1.39)				
pT 2	40	1.26(0.88–2.05)				
pT 3	128	1.20(0.71–1.84)				
pT 4	25	1.12(0.87–1.6)				
pN status			0.094			
pN 0	114	1.25(0.8–2.05)				
pN 1	96	1.16(0.71–1.8)				
pM status			0.828			
pM 0	197	1.21(0.71–2.05)				
pM 1	13	1.16(0.92–1.59)				
pTNM status			0.226			
Stage I	15	1.15(1.03–1.39)				
Stage II	103	1.25(0.8–2.05)				
Stage III	80	1.15(0.71–1.8)				
Stage IV	12	1.22(0.92–1.59)				
Histological differentiation			0.393			
DCIS	3	1.61(1.12–1.67)				
Low	66	1.22(0.8–1.89)				
Moderate	98	1.19(0.71–2.05)				
Well	43	1.17(0.81–1.6)				
Alcohol index			0.815			
Yes	96	1.19(0.71–1.64)				
No	114	1.21(0.8–2.05)				

Prior to use of these serum, informed consent was obtained from each of the patients and healthy participants. All of them provided written informed consent. In our institution, patients were generally followed up every 3 months in the first years, every 6 months for the following 2 years, and annually thereafter for patients without evidence of recurrence. The last follow-up was in October 2015, inform consent and survival status was verified again through direct telecommunication with the patient or their family (performed by The Medical Information Unit in our Cancer Center). This study was approved by the Institute Research Ethics Committee of the Sun Yat-Sen University Cancer Center, Guangzhou, China.

### Laboratory measurements

As part of the physical examination, peripheral blood was collected from the patients between 7 and 8 a.m., before treatment, clotted at room temperature, and centrifuged at 3500 r/min for 8 min. The levels of ApoA-I, Apo-B, HDL-C, LDL-C, TC, and TG were measured using a Hitachi 7600 automatic biochemical analyzer (Hitachi High-Technologies, Tokyo, Japan). ApoA-I and Apo-B were measured using immunoturbidimetry; HDL-C and LDL-C were detected by the selective elimination method (direct method) and selective protection method, respectively; serum TC was measured by the CHOD-PAP method and TC was tested using the GPO-PAP method. All reagents used in this study were provided by Wako Pure Chemical Industries, Japan.

### Statistical analysis

All statistical tests were performed with SPSS 16.0 for Windows software (SPSS, Chicago, IL, USA). OS was calculated between the first diagnosis of ESCC and death, or the date of the last follow-up. Data were expressed as the mean and standard deviation (mean ± SD), and the correlation between ApoA-I and clinical characteristics was assessed using the Mann–Whitney *U* test and *χ*^2^ test. The differences between ESCC patients and healthy donors were compared using the unpaired Student’s *t*-test. Univariate and multivariate analyses of clinical variables were performed using Cox proportional hazards regression models. The results of this survey were analyzed using the Kaplan–Meier survival curves with the log-rank test and proportional hazard model. The correlation between ApoA-I and alcohol index was analyzed using the Spearman rank correlation test. *P* values < 0.05 were regarded as indicating statistically significant differences. All reported *P* values are two sided.

## Results

### Relationship of ApoA-I level with clinical characteristics

This study was a retrospective review that included 210 ESCC patients between January 2007 and July 2009. At the time of the last follow-up, 115 (54.76 %) of the 210 patients had died.

The associations between median serum ApoA-I levels and clinical variables in 210 ESCC patients and controls are presented in Table 1. In the entire cohort, age, sex, pT status, pN status, pM status, pTNM status, histological differentiation or alcohol index had no influence on baseline ApoA-I level both in ESCC patients and controls.

### Pre-therapy serum levels of lipids in ESCC patients and healthy controls

**Fig. 1:**
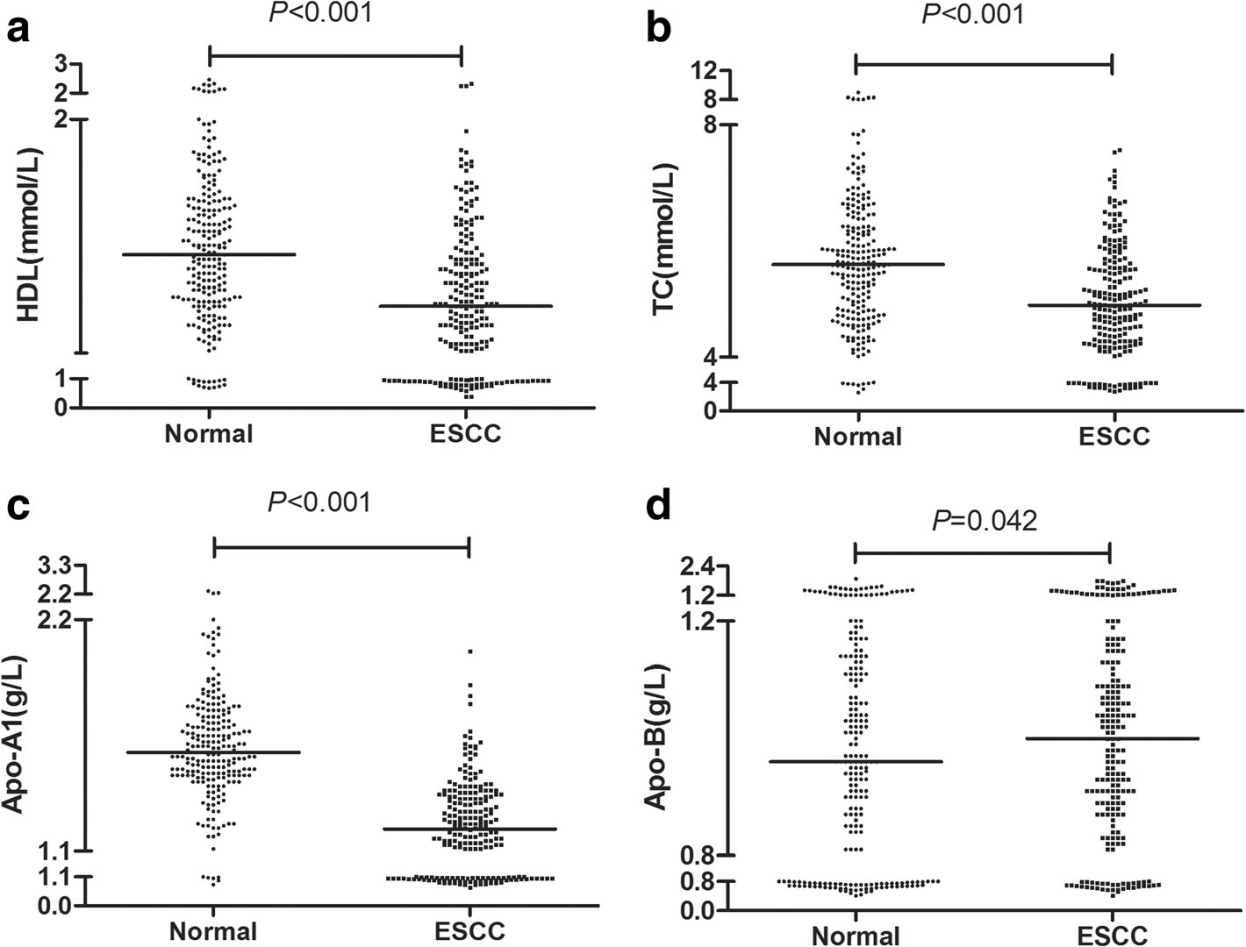
Pre-therapy serum levels of lipids in ESCC patients and healthy controls. The serum HDL-C, TC, ApoA-I, ApoB levels in ESCC patients and healthy controls are plotted as a distribution (**a**, **b**, **c**, **d**)

The levels of lipids and lipoproteins were compared between ESCC patients and healthy controls to investigate lipid abnormalities associated with ESCC (Table 2). The pre-therapy serum levels of ApoA-I (1.22 ± 0.22 mg/dL), HDL-C (1.22 ± 0.32 mg/dL), and TC (4.98 ± 0.95 mg/dL) in ESCC patients were significantly lower than those in the age and sex matched normal controls (ApoA-I: 1.58 ± 0.24 mg/dL; HDL-C: 1.43 ± 0.33 mg/dL; TC: 5.61 ± 1.07 mg/dL), and the level of Apo-B (1.02 ± 0.25 mg/dL) in ESCC patients was higher than that in healthy controls (0.97 ± 0.25 mg/dL). However, there were no significantly differences in TG and LDL-C between ESCC patients (1.26 ± 0.89 and 3.50 ± 0.94) and healthy controls (1.48 ± 1.42 and 3.42 ± 0.98) (Fig. 1).

**Table 2:** The levels of lipids between the ESCC patients and healthy controls

	ESCC		Normal		*p* Value
	Median	(range)	Median	(range)	
ApoA-I(g/L)	1.21	0.71–2.05	1.56	0.8–2.25	**0.000**
ApoB(g/L)	1.00	0.41–1.81	0.96	0.41–1.88	**0.042**
HDL-C(mmol/L)	1.20	0.39–2.34	1.40	0.68–2.47	**0.000**
LDL-C(mmol/L)	3.38	1.32–7.09	3.42	1.17–6.11	0.583
TC(mmol/L)	4.89	2.72–7.57	5.59	2.57–8.92	**0.000**
TG(mmol/L)	1.10	0.38–10.55	1.12	0.4–12.91	0.534

Bold values represent the P-value <0.05, which was considered to be statistically significant

### Univariate and multivariate analysis of clinicopathologic characteristics and lipids in ESCC patients

To determine the prognostic value of pre-treatment lipids and lipoproteins in ESCC patients, the clinical characteristics (including age, gender, tobacco index, alcohol index, pT status, pN status, pM status, pTNM status, and histological differentiation) and lipid levels were subjected to univariate and multivariate analyses. In the univariate analysis, ApoA-I [hazard ratio (HR): 1.541, *p* = 0.016], alcohol index (Yes vs. No, HR: 0.65, *p* = 0.015), pT status (T1-2 vs. T3-4, HR: 0.453, *p* = 0.001), pN status (Yes vs. No, HR: 0.416, *p* = 0.000), pM status (Yes vs. No, HR: 0.401, *p* = 0.003), and pTNM status (I–II vs. III–IV, HR: 0.412, *p* = 0.000) were associated with significantly poor survival (Table 3).

**Table 3:** Univariate and multivariate cox analysis for overall survival in patients with ESCC

Variables	Univariate analysis			Multivariate analysis		
	HR	95 % CI	*p* Value	HR	95 % CI	*p* Value
Sex						
Male vs. Female	1.376	0.918–2.063	0.123			
Age						
<58 vs. ≥58	1.008	0.712–1.428	0.965			
pT status						
T1-2 vs. T3-4	0.453	0.288–0.712	0.001			
pN status						
Yes vs. No	0.416	0.292–0.594	0.000			
pM status						
Yes vs. No	0.401	0.220–0.729	0.003			
pTNM status						
I–II vs. III–IV	0.412	0.289–0.587	0.000	0.427	0.299–0.609	0.000
Histological differentiation						
Differentiated vs. Undifferentiated	0.907	0.227–3.722	0.907			
Tobacco index						
Yes vs. No	0.762	0.528–1.101	0.147			
Alcohol index						
Yes vs. No	0.65	0.458–0.921	0.015	0.688	0.484–0.977	0.037
ApoA-I (g/L)						
<1.21 vs. ≥1.21	1.541	1.082–2.193	0.016	1.519	1.021–2.261	0.039
ApoB(g/L)						
<1.00 vs. ≥1.00	0.927	0.654–1.313	0.669			
HDL-C(mmol/L)						
<1.20 vs. ≥1.20	0.993	0.701–1.407	0.968			
LDL-C(mmol/L)						
<3.38 vs. ≥3.38	0.935	0.659–1.327	0.707			
TC(mmol/L)						
<4.89 vs. ≥4.89	1.041	0.734–1.475	0.824			
TG(g/L)						
<1.10 vs. ≥1.10	1.111	0.784–1.574	0.554			

**Fig. 2:**
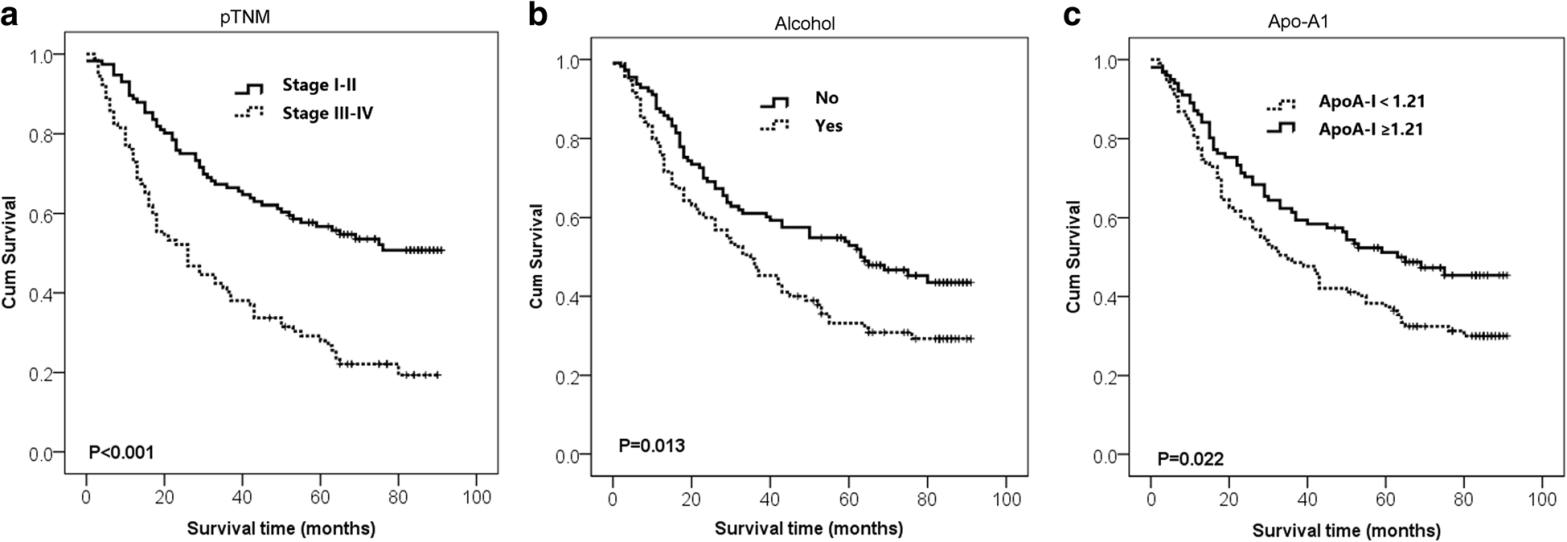
Analysis of 5-year overall survival in ESCC patients. Kaplan–Meier survival curves for overall survival of ESCC patients showing significantly poor survival with higher p TNM status (**a**), alcohol index (**b**) and lower ApoA-I (**c**)

To determine whether these five factors could be used as independent prognostic factors for survival, they were subjected to multivariate analysis. Considering the influence of statistical collinearity, the multivariate model did not include pT status, pN status, or pM status. The results showed that ApoA-I (HR: 1.519, *p* = 0.039), alcohol index (Yes vs. No, HR: 0.688, *p* = 0.037) and pTNM status (I–II vs. III–IV, HR: 0.427, *p* = 0.000) were significant independent predictors of favorable OS. Thus, our findings indicated that serum ApoA-I level before therapy may be a novel independent prognostic factor for ESCC (Fig. 2).

### Kaplan–Meier survival analysis of the levels of independent predictors in ESCC

The Kaplan–Meier method was used to further explore the prognostic significance of ApoA-I level in ESCC, which was used to plot the survival curves. In the whole ESCC cohort, patients with a higher ApoA-I level showed a significantly better 5-year OS than the lower Apo-A1 group. The cumulative 5-year survival rate in the higher ApoA-I group was 48.54 %, whereas it was only 30.84 % in the low ApoA-I group. Furthermore, a high alcohol index and p TNM status were associated with poor 5-year OS in ESCC patients (Fig. 2).

## Discussion

The lack of accurate prognostic biomarkers for patients with ESCC limits therapeutic development, and the long-term survival of these patients remains low. Therefore, the identification of novel prognostic biomarkers for patients with ESCC is critical. In the present study, we compared the levels of serum lipids and lipoproteins (ApoA-I, Apo-B, HDL-C, LDL-C, TC and TG) between ESCC patients and controls. We found that the levels of ApoA-I, HDL-C, and TC were significantly lower, whereas Apo-B was higher in patients with ESCC than in healthy controls. In addition, OS rates were significantly poorer in patients with decreased ApoA-I levels than in patients with high ApoA-I levels, and this was independent of other variables predicting the prognosis of ESCC patients. In addition, alcohol index was an independent prognostic indicator for ESCC, and pTNM status was an independent prognostic factor for poor survival outcome. After adjustment for clinical characteristics, ApoA-I levels were not associated with age, sex, pT status, pN status, pM status, TNM status, histological differentiation or alcohol index.

ApoA-I, a major HDL component in serum, is synthesized in the liver and small intestine, and constitutes approximately 70 % of the apolipoprotein content of HDL particles [21]. Chylomicrons contain ApoA-I, which is quickly transferred to HDL in the bloodstream after being secreted from intestinal enterocytes [22]. ApoA-I is a critical player in reverse cholesterol transport by extracting cholesterol from tissues and transferring it to the liver for its excretion. Cholesterol is a cofactor for lecithin cholesterol acyltransferase, which is responsible for the formation of most plasma cholesteryl esters [23]. ApoA-I is overexpressed in patients with recurrent head and neck squamous cell carcinoma [17]. However, ApoA-I levels are decreased in the serum of patients with pancreatic cancer, colorectal cancer, and ovarian cancer [13, 24, 25]. Low ApoA-I levels are associated with a high cancer risk, specifically with recurrence in breast cancer [8, 26]. Similarly, our study showed that a low ApoA-I level is strongly correlated with poor OS and is an independent prognostic factor for survival (HR: 1.519; 95 % CI: 1.021–2.261).

However, the role of ApoA-I in carcinogenesis is not well understood. In addition to the known functions of ApoA-I, it also possesses anti-inflammatory and antioxidant properties. The group of Farias-Eisner showed that ApoA-I mimetic peptides affect tumor growth and development in mouse models of colon cancer and ovarian cancer [27, 28]. These studies can help establish an association between ApoA-I and ESCC. Lysophospholipids, such as lysophosphatidic acid (LPA), are well-known activators of proliferation in many cancers. A plausible mechanism is that ApoA-I binds LPA to inhibit LPA-induced cell growth. Hazen et al. demonstrated that in the tumor microenvironment, ApoA-I may be work as a potent immunomodulatory agent, altering tumor-associated macrophages from a pro-tumor to an antitumor phenotype [29]. Consistent with these studies, our results showed that patients with low ApoA-I levels had a significantly poor 5-year OS; however, further research is needed to clarify the underlying mechanisms.

In our study, a low alcohol index was a significant independent predictor of favorable OS in ESCC patients (HR: 0.688, 95 % CI: 0.484–0.977). These findings are consistent with previous studies showing a strong association between alcohol consumption and increased risk of ESCC [30, 31]. There is strong epidemiological evidence that consumption of alcoholic beverages increases the risk of cancers of the oral cavity and pharynx, esophagus, and larynx. Accumulating evidence indicates that acetaldehyde is predominantly responsible for the alcohol associated carcinogenesis, since acetaldehyde is carcinogenic, mutagenic, binds to DNA and protein, destroys folate, and results in secondary hyper-regeneration [32]. A number of plausible mechanisms have been proposed, including the relation between alcohol drinking and TC, HDL-C, and apolipoproteins; however, none of these has as yet been firmly established. In the present study, alcohol index was not significantly different between the two categorical ApoA-I groups (Table 1). These results indicated that alcohol consumption was not correlated with ApoA-I concentration. Further studies are needed to clarify these mechanisms.

Although ApoA-I was reported to be a prognostic factor in several malignancies [33], an association between ApoA-I and prognosis in ESCC patients has not been reported to date. In this study, the ApoA-I level was lower in ESCC patients than in normal controls, both ApoA-I and alcohol consumption are independent predictor of OS. Furthermore ApoA-I could function as an independent prognostic factor of ESCC, also it is a common and convenient monitoring index in routine preoperative examination.

## Conclusions

To our knowledge, this is the first retrospective study investigating the relationship between pre-therapy serum lipids and lipoproteins and ESCC. ApoA-I concentration is an independent favorable prognostic factor in ESCC; it shows high reproducibility and can easily be measured in all diagnostic laboratories. Further, we also demonstrated that alcohol consumption influences the survival of patients with ESCC. As this study is a retrospective analysis, it is only valid for generating a hypothesis, and the value of ApoA-I should be validated in large prospective trials.

## Abbreviations

ApoA-I, apolipoprotein A-I; Apo-B, apolipoprotein B; CEA, carcino-embryonic antigen; CYFRA21-1, cytokeratin 19 fragments; ESCC, esophageal squamous cell carcinoma; HDL-C, high-density lipoprotein-cholesterol; LDL-C, low-density lipoprotein-cholesterol; SCC, squamous cell carcinoma antigen; TC, total cholesterol; TG, triglyceride
